# Modelling the impact of stigmatisation of Ebola survivors on the disease transmission dynamics

**DOI:** 10.1038/s41598-023-32040-6

**Published:** 2023-03-24

**Authors:** M. Juga, F. Nyabadza, F. Chirove

**Affiliations:** Department of Mathematics and Applied Mathematics, University of Johannesburg, Auckland Park Campus, Johannesburg, 2006 South Africa

**Keywords:** Diseases, Mathematics and computing

## Abstract

Ebola virus disease (EVD) is one of the most highly stigmatised diseases in any affected country because of the disease’s high infectivity and case fatality rate. Infected individuals and most especially survivors are often stigmatised by their communities for fear of contagion. We propose and analyse a mathematical model to examine the impact of stigmatisation of Ebola survivors on the disease dynamics. The model captures both the internal stigmatisation experienced by infected individuals after witnessing survivors being stigmatised and the external stigmatisation imposed on survivors by their communities. The results obtained from our analysis and simulations show that both internal and external stigma may lead to an increase in the burden of Ebola virus disease by sustaining the number of infected individuals who hide their infection and the number of unsafe burials of deceased Ebola victims. Strategies that seek to put an end to both forms of stigmatisation and promote safe burials will therefore go a long way in averting the EVD burden.

## Introduction

The Ebola virus is a very deadly and highly contagious filovirus that has led to the loss of many lives mainly on the African continent. Due to the high infectivity and case fatality rate of EVD, infected individuals and even survivors are usually stigmatised by their communities in trying to prevent the infection. Many EVD survivors are known to be suffering from short and long-term physical symptoms, mental complications, and stigma as a result of surviving EVD^1–3^.

Stigma constitutes negative attitudes and beliefs that discredit an individual or group of individuals leading to prejudice and societal exclusion^4^. Stigma can lead to experiences and feelings of blame, shame, worthlessness, loneliness, isolation, social exclusion, and discrimination in accessing social amenities and healthcare services^5,6^. Socially undesirable manifestations (prejudice and discrimination) expressed against those with the stigmatising attributes are known as enacted or external stigma whereas the feelings of shame, guilt, or worthlessness experienced as a result of having the stigmatising attributes are referred to as internalised stigma^7^. Stigma in the context of EVD is disconcerting as it originates from structural inadequacies, including poverty, lack of education, and political conflict. These factors combined with cultural practices subsequently influence attitudes, beliefs, and behaviors with respect to disease transmission^3^. It has also been linked to poor adherence to conventional treatment and the utilization of informal or non-integrated forms of health care such as traditional and complementary medicine (TCM)^8,9^. TCM refers to a number of health systems, products, and practices considered to be predominantly outside conventional medical practice and the medical curriculum^10,11^. EVD-related stigma is largely based on community fear that EVD survivors are still contagious. EVD-related stigma has been reported by EVD survivors and their communities in the Democratic Republic of Congo (DRC) $(35\%)$, Guinea $(26\%)$ and Liberia $(3\%),$^12–14^ and may be more common among female than male EVD survivors^15^. Other factors which have been reported as predictors of EVD-related stigma are age, level of education, and having accessed medical care^16^. Liberian research also suggests that EVD survivors are reported to be more likely to experience stigma compared to their close contacts who were not infected with the EVD virus^17^. However, the degree of EVD-related stigma may decline among survivors over time^16,18^. In Sierra Leone, stigmatisation is reported in approximately one third of EVD survivors^19,20^.

Stigmatisation undoubtedly affects EVD transmission dynamics. Since the 2014 EVD outbreak in West Africa which is considered the largest public health emergency in the history of the EVD^21^, a few mathematical models have addressed the impact of various political, economic, social, and human factors, vaccination and treatment on the disease dynamics and have provided insight into the different routes of EVD transmission^22–25^. In certain studies, the provision of hospital beds was estimated to have averted more than 50000 cases in Sierra Leon^26^. Other studies suggest that an increase in media campaigns and the spread of awareness may play a substantial role in decreasing the disease transmission rate^27,28^. It was also suggested in^29^ that political factors like wars and terrorist attacks which continually hinder intervention processes may lead to an increase in the transmission rate. None of the past and present mathematical models have studied the impact of EVD survivor stigmatization on disease transmission.

EVD-related stigma has led to individuals with EVD and EVD survivors being mocked by their communities^30,31^. During the 2000 and 2001 EVD epidemics in Uganda for example, harassment, rejection, and abandonment of individuals with EVD and survivors were common occurrences^32^. Some survivors were also victimized with some being evicted from their homes by their property owners^31,33^, losing their former jobs^34^ and being divorced by their spouses^26,33^. Children were also not spared. There are reports of children orphaned by EVD who remain seronegative but have not been taken up for care by families and communities out of fear of contagion^35^. Some EVD survivors have been prevented from visiting public places such as public toilets and have experienced difficulty in trading commodities at their local market due to a community reluctance to touch their items or money^26,33^. Due to fear of similar treatment, some infected individuals tend to hide their infection and seek informal or non-integrated forms of health care such as traditional and complementary medicine rather than conventional treatment^8,9^. Such changes in human behavior due stigma may affect the disease transmission dynamics. We, therefore, propose a mathematical model which aims to study the impact of EVD survivor-related stigma on the disease transmission rate and the disease eradication process.

## Model formulation

We propose a deterministic model with eight compartments (Susceptible (*S*), Exposed (*E*),  Infected and unstigmatised ($$I_h),$$ Infected and stigmatised $$(I_c),$$ Hospitalised (*H*),  unsafely buried deceased $$(D_u),$$ safely buried deceased $$(D_v),$$ stigmatised survivors $$(R_s),$$ unstigmatised survivors $$(R_n$$)). Susceptible individuals are recruited into the *S* compartment at a constant rate $$\pi .$$ They contact the Ebola virus via physical contact with infectious individuals and dead bodies of Ebola deceased individuals and at a rate $$\lambda$$ and move into the exposed compartment *E*. Depending on the level of stigmatisation experienced by survivors in the community, a proportion of the individuals in the exposed compartment are compelled to remain in the community without seeking hospital care (in the class $$I_C$$) at a survivor stigmatisation-dependent rate $$\epsilon (R_s).$$ The individuals in $$I_c$$ are those who have been exposed to the virus and suspect that they have the virus in their system but decide not to seek hospital care for fear of being stigmatized after recovery. The others move into the compartment of those seeking hospital care, $$I_h$$ at a rate $$\sigma .$$ Therefore,

$$\begin{aligned} \epsilon (R_s)=\;\epsilon _0+\dfrac{\epsilon _1 R_s}{A+R_s}, \end{aligned}$$

where $\epsilon _0$ is the rate of internal stigmatisation, $\epsilon _1$ is the maximum rate of external stigmatisation and $R_s$ is the compartment for stigmatised survivors. The constant *A* is the shape parameter. It determines how fast the effects of reduced stigmatisation can be felt in the case of an outbreak. It is important to note that $0\le \epsilon (R_s)\le 1,$ and therefore, $\epsilon (R_s)$ is defined if and only if $\epsilon _0+\epsilon _1 \le 1.$

Individuals in $I_h$ can either recover at a rate $\theta _1,$ or are hospitalised at a rate $\phi$ or die from the Ebola disease at a rate $\delta _1,$ while those in $I_c$ can either die from the disease at a rate $\delta _3$ or recover at a rate $\theta _4.$ Hospitalised individuals (in compartment H) can also die from Ebola at a rate $\delta _2$ or recover and move into the class $R_s$ at a rate $\theta _3.$ They can also recover and move into the class $R_n$ (class of survivors who are not stigmatised) at a rate $\theta _2.$ We assume that individuals in the classes $I_h$ and $I_c$ who recover without being hospitalised are not stigmatised because they were never diagnosed with the disease, $\rho _1$ is the rate of safe disposal of the dead bodies of infectious individuals who die in the hospital and $\rho _2$ is the rate of unsafe disposal of dead bodies. Individuals in all compartments die from non-EVD related causes at a rate $\mu .$ We assume also that individuals in the class $I_h$ have a naturally reduced transmission compared to those in the stigma class $I_c.$ Individuals who do not disclose their symptoms consequently do not seek hospital care. Infected individuals only start experiencing internalised stigma after they have seen survivors stigmatised by members of their communities. They move into the $I_C$ compartment for fear of being stigmatised after recovery. Therefore, internalised stigmatisation is dependent on external stigmatisation. We assume that all infectious individuals who die in the hospital are safely buried. Also, most of the community burials are unsafe since they are mostly done by the family members of the deceased person. The number of safe community burials is therefore negligible. Hospitalised individuals are kept in controlled environments and are handled by trained medical staff with protective equipment. Safe burials are also done by trained burial teams with protective equipment. We, therefore, assume that the transmissions that occur in hospitals or during safe burials are negligible. The force of infection is thus given by

1 $$\begin{aligned} \lambda = \beta ( D_u+\eta _1I_h +\eta _2I_c), \end{aligned}$$

where $\beta$ is the effective contact rate, $\eta _1$ and $\eta _2$ are the modification parameters for infectiousness. They measure the differences in infectivity of individuals in the classes $I_c$ and $I_h$ compared to the infectivity of those in $D_u.$ The individuals in $I_c$ are hiding their infection, hence we assume that they are more infectious than those in $I_h.$ Therefore $0<\eta _1<\eta _2\le 1.$ We thus have the following model equations.

**Figure 1:**
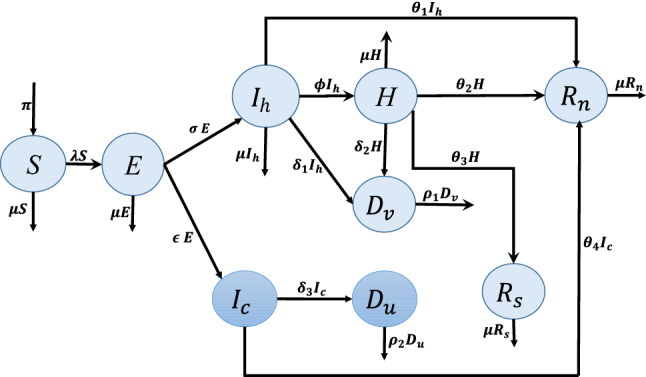
The model diagram for EVD.

2 $$\begin{aligned} \displaystyle \dfrac{dS}{dt}=\; & {} \displaystyle \pi -(\mu +\lambda )S, \end{aligned}$$

3 $$\begin{aligned} \displaystyle \dfrac{dE}{dt}=\; & {} \displaystyle \lambda S-(Q_0+\epsilon (R_s) )E, \end{aligned}$$

4 $$\begin{aligned} \displaystyle \dfrac{dI_{h}}{dt}=\; & {} \sigma E-Q_1I_h, \end{aligned}$$

5 $$\begin{aligned} \displaystyle \dfrac{dI_c}{dt}=\; & {} \epsilon (R_s) E-Q_2I_c, \end{aligned}$$

6 $$\begin{aligned} \displaystyle \dfrac{dH}{dt}=\; & {} \displaystyle \phi I_h-Q_{3}H, \end{aligned}$$

7 $$\begin{aligned} \displaystyle \dfrac{dR_n}{dt}=\; & {} \displaystyle \theta _{1}I_h+\theta _{2}H+\theta _4I_c-\mu R_{n}, \end{aligned}$$

8 $$\begin{aligned} \displaystyle \dfrac{dR_s}{dt}=\; & {} \displaystyle \theta _{3}H-\mu R_{s}, \end{aligned}$$

9 $$\begin{aligned} \displaystyle \dfrac{dD_u}{dt}=\; & {} \displaystyle \delta _{3}I_{c}-\rho _{2}D_{u}, \end{aligned}$$

10 $$\begin{aligned} \displaystyle \dfrac{dD_v}{dt}=\; & {} \displaystyle \delta _{1}I_h+\delta _{2}H-\rho _{1}D_{v}, \end{aligned}$$

where

$$\begin{aligned} \displaystyle Q_0=\mu +\sigma ,~~ Q_{1}=\displaystyle \mu +\theta _{1}+\delta _{1}+\phi ,~~\displaystyle Q_{2}=\displaystyle \mu +\delta _{3}+\theta _{4},~~\displaystyle Q_{3}=\displaystyle \mu +\delta _2+\theta _2+\theta _3, \end{aligned}$$

with initial conditions,

11 $$\begin{aligned} S(0)>0, E(0)\ge 0,I_c(0)\ge 0, I_h(0)\ge 0, H(0)\ge 0, R_n(t)\ge 0, R_s(t)\ge 0, D_u(0)\ge 0, D_v(0)\ge 0, \end{aligned}$$

for all $$t \ge 0$$

## Model analysis

The right-hand side of the system (2)–(10) consists of Lipschitz continuous functions, which is a necessary condition in Picard’s existence theorem^36^. It is therefore sufficient to conclude by Picard’s existence theorem that the solutions of the system (2)–(10) exist and are unique.

We have the following result on the positivity of solutions.

### Theorem 1

The solutions *S*(*t*),  *E*(*t*),  $I_c(t)$, $I_h(t),$ *H*(*t*),  $D_u(t),$ $D_v(t),$ $R_n(t),$ $R_s(t)$ of the system (2)–(10) are non-negative for any given non-negative initial conditions.

### Proof

Let the initial values of the variables of the system of (2)–(10) be non-negative. We prove that the solution *S*(*t*) is non-negative. Assume that there exists a time $t_1$ such that $S(t_1)=0,$ $S'(t_1)<0,$ $S(t)>0,$ $E(t)>0,$ $I_c(t)>0$, $I_h(t)>0,$ $H(t)>0,$ $D_u(t)>0,$ $D_v(t)>0,$ $R_n(t)>0,$ $R_s(t)>0$ for $0<t<t_1.$ From (2), we have

$$\begin{aligned} \dfrac{dS(t_1)}{dt} =\pi >0. \end{aligned}$$

This contradicts the assumption that $S'(t_1)<0.$ Therefore *S*(*t*) is positive.

Similarly, *E*(*t*),  $I_c(t)$, $I_h(t),$ *H*(*t*),  $D_u(t),$ $D_v(t),$ $R_n(t),$ $R_s(t)$ remain non-negative for any given non-negative initial conditions. $\square$

We now prove the following theorem on the boundedness of the solutions.

### Theorem 2

Given the initial conditions (11), the solutions of the system (2)–(10) will always be non-negative and bounded in the positively invariant region $\Omega$ given by

$$\Omega =\bigg \lbrace (S,E, I_{h},l_{c},H,R_n,R_s,D_u,D_v)\in \mathbb {R}^{9}_{+}:N(t)\le \displaystyle \frac{\pi }{\mu }, D_u\le \dfrac{\delta _3\pi }{\mu \rho _2},~ D_v\le \dfrac{(\delta _1+\delta _2)\pi }{\mu \rho _1} \bigg \rbrace .$$

### Proof

Given $N(t) =S(t)+E(t)+I_{h}(t)+l_{c}(t)+H(t)+R_s(t)+R_n(t),$ adding Eqs. (2)–(8) we obtain

$$\begin{aligned} \dfrac{dN}{dt}= & {} \pi -\mu N -(\delta _1I_h+\delta _3I_c+\delta _2H)\\\le & {} \pi -\mu N. \end{aligned}$$

Separating variables and solving for N(t), we obtain

$$\begin{aligned} N(t)\le \dfrac{\pi }{\mu }-\bigg (\dfrac{\pi }{\mu }-N_0\bigg )\exp {(-\mu t)}. \end{aligned}$$

Therefore,

$$\begin{aligned} \limsup _{t\longrightarrow \infty }N(t)\le \dfrac{\pi }{\mu }. \end{aligned}$$

Since *N*(*t*) is equal to the sum of the state variables, we have that each of the individual state variables is less than or equal to $\dfrac{\pi }{\mu }.$

From Eq. (9),

$$\begin{aligned} \dfrac{dD_u}{dt}= & {} \delta _3I_c-\rho _2D_u\\\le & {} \delta _3\dfrac{\pi }{\mu }-\rho _2D_u. \end{aligned}$$

The solution of this differential inequality can be obtained using a suitable integrating factor so that

$$\begin{aligned} D_u(t)\le \dfrac{\pi \delta _3}{\mu \rho _2}-\bigg (\dfrac{\pi \delta _3}{\mu \rho _2}+D_{u0}\bigg )\exp {(-\rho _2t)}. \end{aligned}$$

Therefore,

$$\begin{aligned} \limsup _{t\longrightarrow \infty } D_u(t)\le \dfrac{\pi \delta _3}{\mu \rho _2} \end{aligned}$$

Similarly,

$$\begin{aligned} D_v(t)\le \dfrac{\pi (\delta _1+\delta _2)}{\mu \rho _1}-\bigg (\dfrac{\pi (\delta _1+\delta _2)}{\mu \rho _1}+D_{v0}\bigg )\exp {(-\rho _1t)}, \end{aligned}$$

and

$$\begin{aligned} \limsup _{t\longrightarrow \infty } D_v(t)\le \dfrac{\pi (\delta _1+\delta _2)}{\mu \rho _1}. \end{aligned}$$

We can conclude that the solutions are all bounded and $\Omega$ is positively invariant and attracts all positive solutions of the system (2)–(10). $\square$

## Model equilibrium points and stability analysis

In this section, we obtain the equilibrium points of the model (2)–(10) by setting the right-hand side of the system to zero so that,

12 $$\begin{aligned} \displaystyle \pi -(\mu +\lambda ^*)S^*=\;& {} 0, \end{aligned}$$

13 $$\begin{aligned} \displaystyle \lambda ^* S^*-(Q_0+\epsilon (R^*_s)) E^*=\;& {} 0, \end{aligned}$$

14 $$\begin{aligned} \displaystyle \sigma E^*-Q_1I_h^{*}=\;& {} 0, \end{aligned}$$

15 $$\begin{aligned} \displaystyle \epsilon (R^*_s) E^*-Q_2I_c^{*}=\;& {} 0, \end{aligned}$$

16 $$\begin{aligned} \displaystyle \phi I_h^{*}-Q_{3}H^*=\;& {} 0, \end{aligned}$$

17 $$\begin{aligned} \displaystyle \theta _{1}I_h^{*}+\theta _{2}H^*+\theta _4I_c-\mu R_{n}^{*}=\;& {} 0, \end{aligned}$$

18 $$\begin{aligned} \displaystyle \theta _{3}H^*-\mu R_{s}^{*}=\;& {} 0, \end{aligned}$$

19 $$\begin{aligned} \displaystyle \delta _{3}I_{c}^{*}-\rho _{2}D_{u}^{*}=\;& {} 0, \end{aligned}$$

20 $$\begin{aligned} \displaystyle \delta _{1}I_h^{*}+\delta _{2}H^*-\rho _{1}D_{v}^{*}=\;& {} 0. \end{aligned}$$

From (14), (16), (17), (18), (19), (20), we obtain

$$\begin{aligned} \begin{array}{lll} E^*=\psi _1I^*_{h},&{} H^*=\psi _2I^*_{h}, &{}R^*_{n}=\psi _3I^*_{h}+ \psi _4I*_{c}, \\ D^*_{u}=\psi _6I^*_{c},&{}D^*_{v}=\psi _7I^*_{h},&{} R^*_{s}=\psi _5I^*_{h}, \end{array} \end{aligned}$$

where

$$\begin{aligned} \begin{array}{lll} \psi _1=\dfrac{Q_1}{\sigma },&{}\psi _2=\dfrac{\phi }{Q_3},&{}\psi _3=\dfrac{\theta _1+\theta _2\psi _2}{\mu }, ~~~\psi _4=\dfrac{\theta _3}{\mu }, \\ \\ \psi _5=\dfrac{\theta _3\psi _2}{\mu },&{} \psi _6=\dfrac{\delta _3}{\rho _2},&{}\psi _7=\dfrac{\delta _1+\delta _2\psi _2}{\rho _1}. \end{array} \end{aligned}$$

Replacing the expression for $D_u^{*}$ in (1), we obtain an expression for $\lambda ^*$ as

21 $$\begin{aligned} \lambda ^*=\beta (\eta _1I^*_{h}+\psi _8I^*_{c}), \end{aligned}$$

where $\psi _8=\psi _6+\eta _2.$ Substituting (21) into (12) and the expression for $E^*$ into (15), we get

$$\begin{aligned} S^*=\dfrac{\pi }{\mu +\beta \eta _1I_h^{*}+\beta \psi _8I_c^{*}} \end{aligned}$$

and

$$\begin{aligned} I_c^{*}=\dfrac{\psi _1(A\epsilon _0+\psi _{5}(\epsilon _0+\epsilon _1)I_h^{*})}{Q_2(A+\psi _5I_h^{*})}I_h^{*}. \end{aligned}$$

Substituting the expressions for $I_c^{*}$ and $E^*$ into (13) results in the $4^{th}$ degree equation

$$\begin{aligned} I_h^{*}(a_3I_h^{*3}+a_2I_h^{*2}+a_1I_h^{*}+a_0)=0, \end{aligned}$$

which yields

22 $$\begin{aligned} I_h^{*}=0 \end{aligned}$$

or

23 $$\begin{aligned} a_3I_h^{*3}+a_2I_h^{*2}+a_1I_h^{*}+a_0=0, \end{aligned}$$

where

$$\begin{aligned} \begin{array}{lll} a_3&{}=&{}\beta \psi _1\psi _5^2 \bigg [Q_2\eta _1(Q_0+\epsilon _0+\epsilon _1)+\psi _1\psi _8(\epsilon _0(Q_0+\epsilon _0)+\epsilon _1(Q_0+2\epsilon _1)+\epsilon _1^2) \bigg ],\\ \\ a_2&{}=&{}-\bigg [\dfrac{Q_1Q_2\psi _5^2(Q_0+\epsilon _0)(1-R_0))}{\sigma \pi }-A\beta \psi _1\eta _1(2(Q_0+\epsilon _0)+Q_2\epsilon _1)\\ {} &{}&{}+\psi _1\psi _5^2\epsilon _1(\pi \beta \psi _8+\mu Q_2)+2A\beta \psi _1^2\psi _5\psi _8(\epsilon _0(Q_0+\epsilon _0)+\epsilon _1)\bigg ],\\ \\ a_1&{}=&{}-A^2(Q_0+\epsilon _0)\bigg [\dfrac{2AQ_1Q_2\psi _5(1-R_0))}{\sigma }- \beta \psi _1(Q_2\eta _1+\psi _1\psi _8\epsilon _0)+A\epsilon _1\psi _5\psi _1(\mu Q_2+\pi \beta \psi _5\psi _8) \bigg ],\\ \\ a_0&{}=&{}\dfrac{A^2Q_1Q_2\rho _2(Q_0+\epsilon _0 )}{\sigma }(1-R_0), \end{array} \end{aligned}$$

with

$$\begin{aligned} R_0=\dfrac{\pi }{\mu }\beta \bigg [\dfrac{Q_2\eta _1\rho _2\sigma +Q_1\epsilon _0(\delta _3+\eta _2\rho _2)}{Q_1Q_2(Q_0+\epsilon _0 )\rho _2} \bigg ]. \end{aligned}$$

When $I_h^{*}=0,$ we have that $E^*=I_c^{*}=H^*=R_n^{*}=R_s^{*}=D_u^{*}=D_v^{*}=0$ and $S^*=\dfrac{\pi }{\mu }.$ This gives the disease-free equilibrium point (DFE),

$$\begin{aligned} E_0=\bigg (S^0,E^0,I_h^{0},I_c^{0},R_n^{0},R_s^{0},D_u^{0},D_v^{0}\bigg )=\bigg (\dfrac{\pi }{\mu },0,0,0,0,0,0,0,0\bigg ). \end{aligned}$$

We discuss the existence of positive solutions to Eq. (23) using Descarte’s rule of signs, Euclid’s algorithm, and Sturm’s sequences^37^. Table 1 summarises Descartes’ rule of signs on Eq. (23).

**Table 1:** Number of possible positive roots of Eq. (23) using Descartes’ rule of signs.

case	$a_3$	$a_2$	$a_1$	$a_0$	possible positive roots	$R_0$ condition
(i)	$+$	+	+	+	0	$R_0<1$
(ii)	$+$	+	+	−	1	$R_0>1$
(iii)	$+$	+	−	+	2 or 0	$R_0<1$
(iv)	$+$	+	−	−	1	$R_0>1$
(v)	$+$	−	+	+	2 or 0	$R_0<1$
(vi)	$+$	−	+	−	3 or 1	$R_0>1$
(vii)	$+$	−	−	+	2 or 0	$R_0<1$
(viii)	$+$	−	−	−	1	$R_0>1$

Table 1 shows the existence of a unique endemic equilibrium state in cases (i), (v), and (vii) whenever $a_2>0$ or $a_2<0,$ $a_1<0$ and $R_0>1.$ We use Sturm’s sequences and Euclid’s algorithm to determine the exact number of positive roots in the remaining cases. We develop the Sturm’s sequence polynomials $S_i(I_h^{*}),$ $i=1,2,3,4$ as follows:

We set $S_1(I_h^{*})$ equal to the left-hand side of Eq. (23). $S_2(I_h^{*})$ equals the derivative of $S_1(I_h^{*})$ with respect to $I_h^{*}.$ $S_3(I_h^{*})$ is the negative remainder obtained upon application of Euclid’s algorithm on $S_1$ and $S_2$ while $S_4(I_h^{*})$ is the negative constant remainder obtained by applying Euclid’s algorithm on $S_2$ and $S_3$ so that

$$\begin{aligned} S_1(I_h^{*})=\:& {} a_3I_h^{*3}+a_2I_h^{*2}+a_1I_h^{*}+a_0,\\ S_2(I_h^{*})=\:& {} 3a_3I_h^{*2}+2a_2I_h^{*}+a_1,\\ S_3(I_h^{*})=\:& {} (2a^2_{2}-6a_1a_3)I_h^{*}+a_2a_1-9a_0a_3,\\ S_4(I_h^{*})=\:& {} \dfrac{(9a_0a_3-a_2a_1)(15a_2a_1a_3-27a_0a_3^{2}-4a_2^{3})-a_1(6a_1a_3-2a_2^{2})^2}{(6a_1a_3-2a_2^{2})^2}. \end{aligned}$$

According to Sturm’s theorem^37^, we choose any two real numbers $c_1 = 0$ and $c_2,$ (significantly large and positive). The number of non-repeated real positive roots between $c_1$ and $c_2$ is the difference between the number of sign changes in the Sturm sequence when $I_h^{*} = c_1$ and the number of sign changes when $I_h^{*} = c_2.$ The sturm sequences evaluated at $c_1$ and $c_2$ are given on (24) and (25) respectively.

24 $$\begin{aligned} S_1(0)= & {} a_0,~ S_2(0) = a_1,~ S_3(0) = a_2a_1-9a_0a_3,~ S_4(0) = S_4(I_h^{*}). \end{aligned}$$

25 $$\begin{aligned} S_1(c_2)= & {} a_3c^3_{2}, ~S_2(c_2) = +3a_3c_2^{2},~ S_3(c_2) =(2a^2_{2}-6a_1a_3)c_2,~S_4(c_2) = S_4(I_h^{*}). \end{aligned}$$

### Theorem 3

Consider the Sturm sequences evaluated at $c_1$ and $c_2$ as shown in (24) and (25). we have the following four possibilities.

**Case 1**: If $a_2 > 0,$ $a_1 < 0,$ $R_0 > 1$ and either

$$\begin{aligned} \begin{array}{lllll} (a)&{} S_3(0)> 0,&{} S_4(0)> 0,&{}S_3(c_2)> 0&{}S_4(c_2)>0,\\ (b)&{} S_3(0)>0,&{} S_4(0)>0,&{}S_3(c_2)<0 &{}S_4(c_2)>0,\\ (c)&{} S_3(0)> 0,&{} S_4(0)< 0,&{}S_3(c_2)> 0&{}S_4(c_2)<0, \\ (d)&{} S_3(0)< 0,&{} S_4(0)>0,&{}S_3(c_2)> 0&{}S_4(c_2)>0,\\ (e)&{} S_3(0)< 0,&{} S_4(0)> 0,&{}S_3(c_2)< 0&{}S_4(c_2)>0 ~~~ \textrm{or}\\ (f ) &{}S_3(0)< 0,&{} S_4(0)< 0,&{}S_3(c_2)< 0,&{}S_4(c_2) < 0,\\ \end{array} \end{aligned}$$

then the cubic equation has exactly one positive root.

**Case 2**: If $a_2 > 0,$ $a_1 > 0,$ $R_0 < 1$ and either

$$\begin{aligned} \begin{array}{lllll} (a)&{} S_3(0)> 0,&{} S_4(0)> 0,&{}S_3(c_2)> 0&{}S_4(c_2)>0,\\ (b)&{} S_3(0)< 0,&{} S_4(0)< 0,&{}S_3(c_2)>0 &{}S_4(c_2)<0,\\ (c)&{} S_3(0)< 0,&{} S_4(0)< 0,&{}S_3(c_2) < 0&{}S_4(c_2)>0 \\ \end{array} \end{aligned}$$

or

If $a_2 > 0,$ $a_1 < 0,$ $R_0 < 1,$ and either

$$\begin{aligned} \begin{array}{llll} (a)&{} S_4(0)> 0,&{}S_3(c_2)> 0&{}S_4(c_2)>0,\\ (b)&{}S_4(0)> 0,&{}S_3(c_2)<0 &{}S_4(c_2)>0,\\ (c)&{} S_4(0)< 0,&{}S_3(c_2)<0&{}S_4(c_2)<0 \\ \end{array} \end{aligned}$$

   or

If $$a_2 < 0,$$ $$a_1 >0,$$ $$R_0 < 1,$$ and $$S_3(0)<0,$$ $$S_4(0)<0,$$ $$S_3(c_2)>0,$$ $$S_4(c_2)<0,$$

then the cubic Eq. (23) has no positive root.

**Case 3**: If $a_2 > 0,$ $a_1 >0,$ $R_0 < 1,$ or $a_2<0,$ $a_1>0,$ $R_0<1$ and $S_4(0) > 0,$ $S_3(c_2) > 0,$ $S_4(c_2)>0,$ then, the cubic Eq. (23) has exactly 2 positive roots.

**Case 4**: If $a_2 > 0,$ $a_1 <0,$ $R_0 > 1,$ and $S_4(0) < 0,$ $S_3(c_2) < 0$ $S_4(c_2)<0,$ then, the cubic Eq. (23) has exactly 3 positive roots.

The results obtained from Sturm’s theorem indicate that the Eq. (23) can either have 0, 1, 2, or 3 roots depending on the signs of its coefficients. The case of zero roots (second case of Theorem 3) corresponds to the case where all the roots are either negative or a combination of negative and complex roots, which represents a situation where the system has no endemic equilibrium point but only a DFE. the first case of Theorem 3 may suggest the case of a forward bifurcation where the system has exactly one endemic equilibrium. The third case shows two endemic equilibrium points coexisting with the DFE, which may suggest the possibility of a backward bifurcation in the system. The fourth case is a case of three endemic equilibrium points in the system.

## Numerical simulations

Here, we perform numerical simulations on the model (2)–(10) to assess the role of stigma in the transmission dynamics of EVD in a population. In particular, the impact of Ebola survivor stigmatisation as well as the stigmatisation of infected individuals on the proportion of infected individuals who seek hospital care, the number of Ebola deceased with safe and unsafe burials will be investigated and their contributions to the EVD burden of the country will be quantified.

### Model parameters

The numerical values (or ranges) of the model parameters used in the simulations are given in Table 2. While some of the parameter values were obtained from existing literature, others were estimated or fitted. For instance, the demographic parameter $\mu$ is estimated as $\mu =\frac{1}{60\times 52}$ per week, where 60 yrs is the average lifespan in the DRC^38^. The parameter $\pi$ is then estimated as follows: since the total estimated population of the North and South Kivu provinces for the year 2020 is 15213800^39^, we assume that the total limiting population in the absence of disease $\frac{\pi }{\mu }$ is 15213800,  so that $\pi =253563.3/52$ per week.

**Figure 2:**
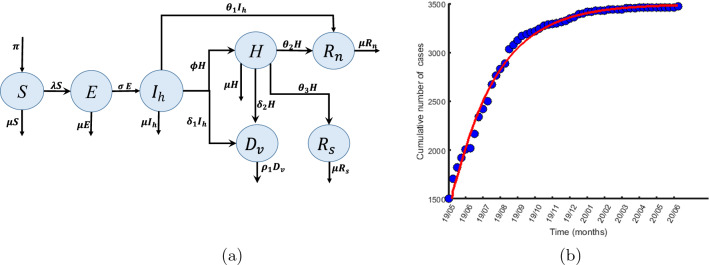
(**a**) Diagram of the model without stigma (**b**) Data fitting of the model in (**a**) to cumulative EVD cases. The data is for the 2019/2020 EVD outbreaks in the DRC extracted from the WHO website, specifically from May 2019 to June 2020^42^.

The fitted parameters were obtained by fitting the model in Fig. 2 (the model without the stigma parameters) to the weekly EVD data for DRC (North and South Kivu provinces) from May 2019 to June 2020^40^, (see Fig. 2). It is important to note that there is no existing data for stigmatised Ebola-infected individuals. The data in^40^ used for the fitting is clinical data obtained after the individuals had been tested for Ebola. However, our model considers the stigmatized infected individuals (those in the $I_c$ compartment) as those who because of stigma, never showed up at any hospital or testing center to be tested for the disease but remained in the community or sought health care from TCM practitioners. The data in^40^ is therefore for individuals in the $I_h$ compartment only. We thus fit the data to the reduced model (the model in Fig. 2 obtained by withdrawing the parameters $\epsilon ,$ $\delta _3,$ $\theta _4,$ $\rho _2.$) without stigma instead of the model (2)–(10). After the fitting, we reasonably estimate some of the stigma parameters that were withdrawn, assume others, and then carry out global sensitivity analysis on the entire parameter space especially targeting the withdrawn parameters. For instance, the individuals in $I_h$ (who are not stigmatised) go to the hospital as soon as they start having symptoms of the disease. They seek medical care and reduce their chances of dying from Ebola. However, those in $I_c$ who are stigmatised hide their infection, hence, they are more likely to die of the disease than those in $I_h.$ We therefore assume that $\delta _3>\delta _1.$ Since the fitted value of $\delta _1$ is 0.42/*week*,  we thus choose the value of $\delta _3$ to be 0.54/*week*. Also, infected individuals in the hospital are in controlled environments undergoing treatment, they are more likely to recover than those in $I_c$ who hide their infection and refuse formal treatment. Hence we assume that $\theta _2>\theta _4$ and choose $\theta _4$ to be 0.01/*week*. Similarly, we assume that $\rho _2>\rho _1,$ and choose $\rho _2=0087.$ Since Sierra Leon suffered Ebola outbreaks of a similar structure as those of the DRC, we, therefore, adopt the value of $\epsilon _0$ for sierra Leon estimated by James et al in^3^ to be in the range [0.15,1.69]. We thus choose $\epsilon _0=0.24,$ and $\epsilon _1=0.45$ so that $\epsilon _0+\epsilon _1\le 1.$ The parameter values are given in Table 2.

**Table 2:** Estimated parameter values for model (2)–(10).

Parameter	Description	Value	Reference
$\pi$	Recruitment rate	4876.2 people/week	Estimated
$\beta$	Contact rate	0.045/people$\times$ week	Fitted
$\mu$	Natural mortality rate	0.0003/week	Estimated
$\delta _{3}$	Disease related death of the infected in $I_c$	0.54/week	Estimated
$\delta _{1}$	Disease related death of the infected in $I_h$	0.42/*week*	Fitted
$\delta _{2}$	Disease related death of the hospitalized	0.2/*week*	Fitted
$\theta _{2}$	Rate of recovery into $R_n$	0.8/week	Fitted
$\theta _{1}$	Rate of recovery of the infected in $I_h$	0.031/week	Fitted
$\theta _{3}$	Rate of recovery into $R_s$	0.5/week	Fitted
$\theta _{4}$	Rate of recovery of the infected in $I_c$	0.01/week	Estimated
$\rho _1$	Rate of save disposal of dead bodies	0.005/week	fitted
$\rho _2$	Rate of unsafe disposal of dead bodies	0.0087/week	Assumed
$\epsilon _0$	internal stigmatization rate	0.24/week	Estimated
$\epsilon _1$	maximum stigmatization rate	0.45/week	Estimated
$\phi$	Rate of hospitalization of the infectious	0.041/week	^41^
$\sigma$	Progression rate from *E* to $I_h$	0.0028/week	Fitted
$\eta _1$	Modification parameter $(I_h)$	0.09	^40^
$\eta _2$	Modification parameter $(I_c)$	0.2	Assumed
*A*	Shape parameter	20	Assumed

### Sensitivity analysis

**Figure 3:**
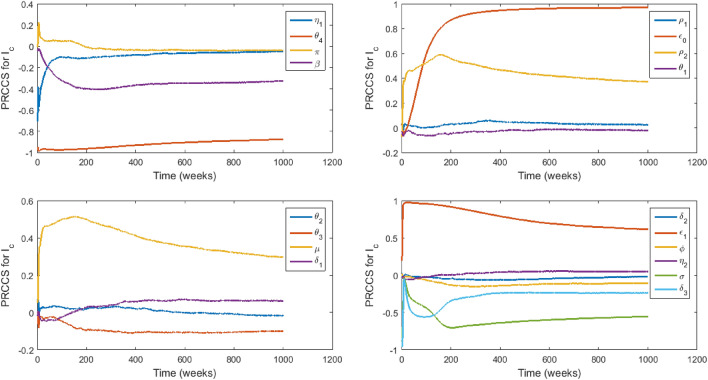
PRCC values of model (2)–(10) with $I_c$ as the response function.

**Figure 4:**
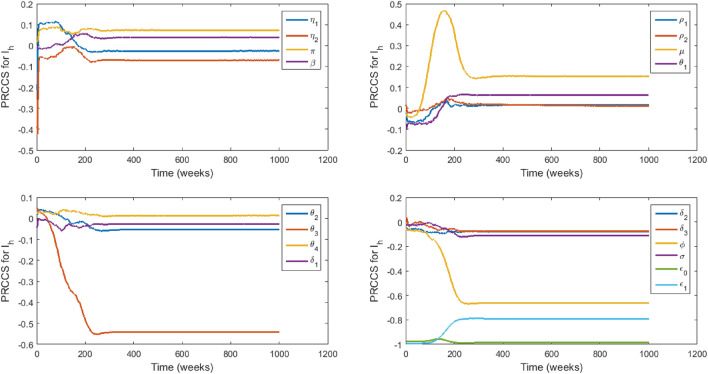
PRCC values of model (2)–(10) with $I_h$ as the response function.

**Figure 5:**
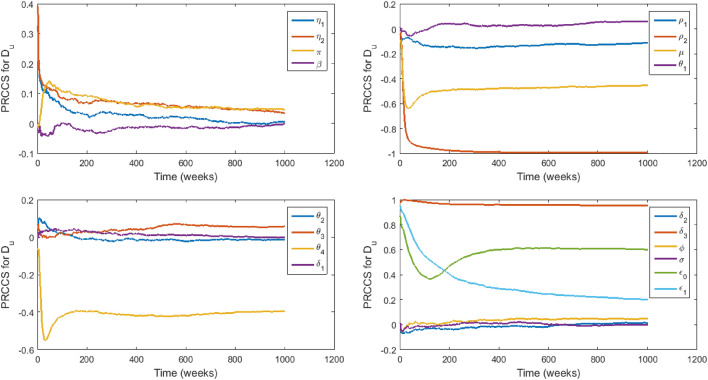
PRCC values of model (2)–(10) with *Du* as the response function.

We carry out sensitivity analysis^43,44^ on all of the model parameters with specific state variables ($I_c,$ $I_h,$ $D_u$) as the response functions to determine which of the parameters have the most significant impact on the outcome of the numerical simulations of the model. The specific state variables chosen are the infectious classes that play a more significant role in the disease transmission dynamics and therefore capture the model objective which is to evaluate the impact of stigmatization on EVD transmission. Figures 3, 4 and 5 show the partial rank correlation coefficients (PRCC) over time. We use the PRCC’s to identify which parameters are key contributors in predicting the changes in the number of individuals in the classes $I_c,$ $I_h,$ and $D_u$ over time. The magnitude of the PRCC indicates the importance of the uncertainty in estimating the value of the specific variable, while the sign of the PRCC indicates the qualitative relationship between the parameter and the state variable. The most significant parameters in Fig. 3 are $\epsilon _0,$ $\epsilon _1,$ $\sigma ,$ $\delta _3$ and $\mu .$ $\epsilon _0$ and $\epsilon _1$ are positively strongly correlated to $I_c,$ which indicates that a little increase in stigmatisation will lead to a significant increase in the number of infected individuals who hide their infection and refuse to seek hospital care. This may lead to an increase in the disease transmission, creating a greater EVD burden on the affected community. Therefore, since $\epsilon$ depends on $\epsilon _0$ and $\epsilon _1,$ the uncertainty in estimating the value of $\epsilon$ is very critical in affecting the prediction imprecision of the number of individuals in $I_c$ and in the control of EVD. The parameters $\sigma ,$ $\phi ,$ $\beta$, $\pi ,$ $\eta _1,$ $\eta _2,$ $\theta _4,$ $\theta _3,$ and $\delta _3$ are negatively correlated to $I_c$ but $\delta _1,$ $\beta$, $\pi ,$ $\eta _1$ and $\eta _2$ are of lesser importance $(-0.35<PRCC\le -0.35)$ in contributing to prediction imprecision^43^. The signs and sizes of the PRCCs of the parameters $\sigma ,$ $\delta _3$ and $\theta _4$ indicate that the uncertainty in estimating their values is also critical in affecting the prediction imprecision of the number of individuals in $I_c$ and an increase in their values will lead to a decrease in the number of individuals in $I_c.$ Also, the uncertainty in predicting the value of $\delta _3$ is crucial because an increase in $\delta _3$ will promote unsafe burials and hence the creation of more new infections during the burial process. However, this can be countered by higher values of $\theta _4$ since the prevalence of stigmatised survivors will cause a fall in the stigmatised infectious population and hence a fall in the disease transmission. The correlation of the parameters to the remaining state variables can be explained in a similar manner.

### The impact of Stigma in the EVD transmission dynamics

**Figure 6:**
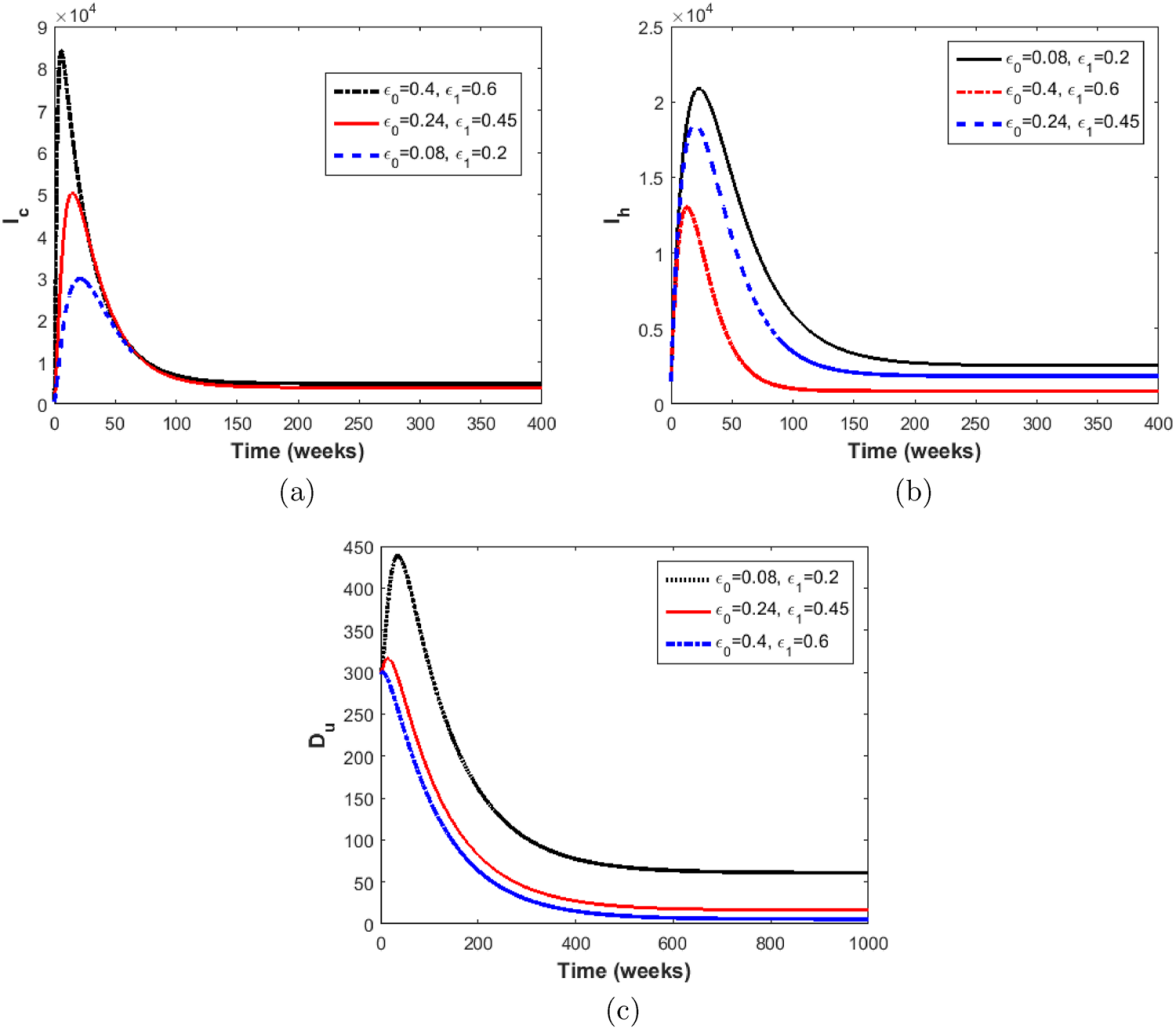
Simulations of the model (2)–(10) with different values of $\epsilon _0$ and $\epsilon _1$ (**a**) Number of infected stigmatised individuals, (**b**) the number of infected unstigmatised individuals, (c) Number of dead bodies that are unsafely buried.

**Figure 7:**
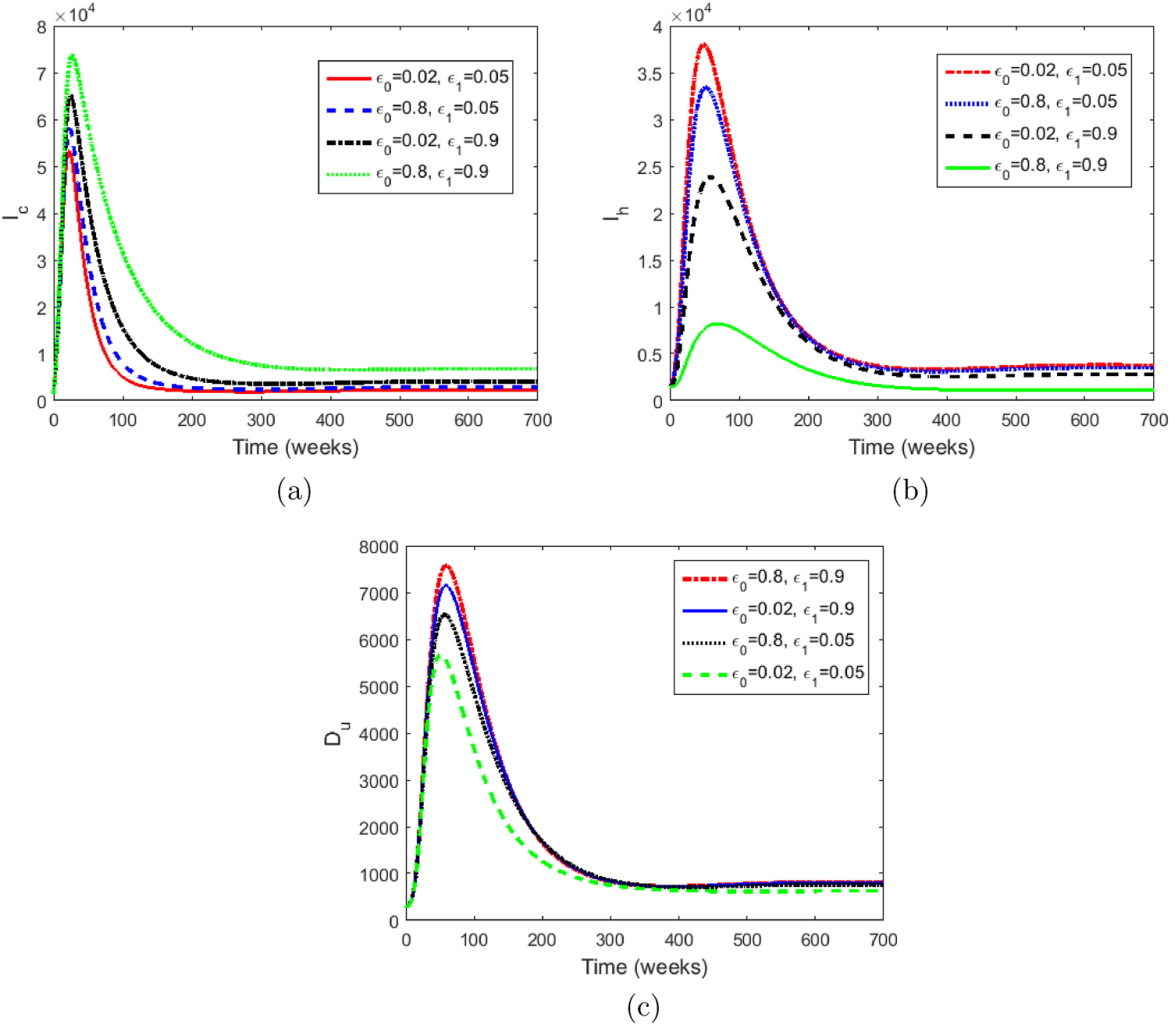
Simulations of the model (2)–(10) with different values of $\epsilon _0$ and $\epsilon _1.$ each graph represents either the case where both epsilons are high, or both low, or one is high, and the other is low. (**a**) The number of infected stigmatised individuals, (**b**) the number of infected unstigmatised individuals, and (**c**) the number of dead bodies that are unsafely buried.

We assess the effect of the stigma parameters ($\epsilon _0$ and $\epsilon _1$) by simulating the model (2)–(10) using the parameters in Table 2 and various values of $\epsilon _0$ and $\epsilon _1$ (the parameters that models stigma). The initial conditions used in the simulations are $S=15182200,$ $E=20000,$ $I_h=1500,$ $I_c=1200,$ $H= 800,$ $D_u=300,$ $D_v= 700.$ The results obtained are shown in Figs. 6, and 7.

Figure 6a depict an increase in the number of infected individuals who hide their infection and refuse to seek hospital care with increasing values of $\epsilon _0$ and $\epsilon _1.$ $\epsilon _0=0.24$ and $\epsilon _1=0.45$ are the baseline stigma values (estimated). They represent the average values of internal and external stigma respectively for which EVD will persist in the population. In Fig. 6a, we observe a slight increase in the infected stigmatised population as the stigma parameter values are raised above the baseline values ($\epsilon _0=0.4$ and $\epsilon _1=0.6$), and a fall in the infectious stigmatised population when the values are decreased below the baseline values. Also, increasing $\epsilon _0$ and $\epsilon _1$ leads to an increase in the reproduction number from $R_0= 2.62$ to $R_0=3.11.$ This shows that a greater EVD burden will be recorded in such a community if anti-stigmatisation strategies are not implemented to reduce or prevent EVD survivors stigmatisation. Similar results were obtained for the infected unstigmatised and deceased individuals. In Fig. 6c we observe that increasing $\epsilon _0$ and $\epsilon _1$ leads to more unsafe burials than safe burials. This has a devastating impact on the disease dynamics. Since the dead bodies of Ebola deceased individuals are more infectious than the infected who are alive, more unsafe burials may lead to a drastic increase in disease infections.

Figure 7 shows the changes that occur in the infectious population if one or both of $\epsilon _0$ and $\epsilon _1$ are increased and or decreased. In Fig. 7a We observe that for values of $\epsilon _0$ and $\epsilon _1$ lower than the baseline values ( $\epsilon _0=0.24$ and $\epsilon _1=0.45$), the number of infectious stigmatised individuals decrease. However, an increase in the value of $\epsilon _1$ above the baseline value, keeping the value of $\epsilon _0$ below the baseline value results in a slightly greater increase in the number of infectious stigmatised individuals than the case when $\epsilon _0$ is high, and $\epsilon _1$ is low. This indicates that $\epsilon _1$ has a greater impact on disease transmission than $\epsilon _0.$ On the other hand, we see the combined effect of $\epsilon _0$ and $\epsilon _1$ in the much greater increase in the number of infected individuals when both values are high. Anti-stigmatisation measures are therefore recommended for disease control. Similar explanations can be made for Fig. 7b and c.

**Figure 8:**
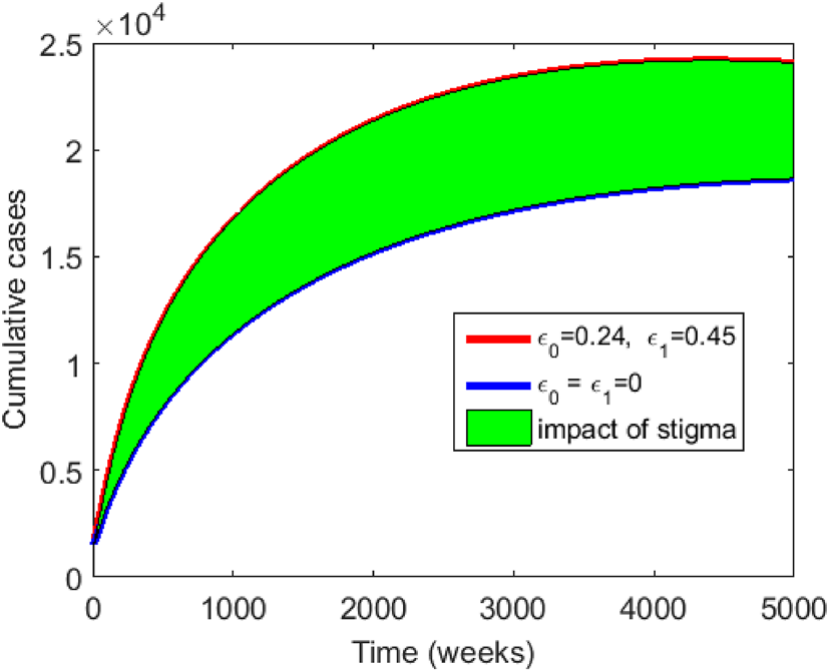
Graph of cumulative EVD cases without stigma in blue and with stigma in red.

Figure 8 shows the graphs of the infected population for the model without stigma in Fig. 2 and the model with stigma in Fig. 1. The graph in red represents the cumulative cases in the presence of stigmatisation while the one in blue represents the cumulative cases in the absence of stigma. The area of the shaded region depicts the impact of stigma on the infection rate. The observed fall in the number of infected individuals in the absence of stigmatisation indicates the importance of targeting control measures that focus on reducing EVD-related stigma if disease eradication must be attained.

## Conclusion

EVD is highly infectious, thus, infected individuals are usually kept in confined environments and handled by highly trained medical staff with protective equipment to reduce the disease transmission rate. Due to the fear of being stigmatised after recovery, some infected individuals hide their infection and refuse to seek hospital care. This leads to an increase in the disease transmission rate as well as an increase in the rate of unsafe burials. Stigmatisation thus has an impact on EVD transmission dynamics which this paper investigated. We developed a mathematical model which uses a saturating stigmatisation function to capture the role of both internal and external stigma.

The model has a stigma-dependent basic reproduction number, $R_0,$ a locally stable disease-free equilibrium, Zero or two endemic equilibrium points when $R_0<1,$ and one or three endemic equilibrium points when $R_0>1.$ Since there is no available data for stigmatised ebola cases, we carried out parameter estimation by withdrawing the stigma parameters from the model by fitting the resulting model to data and reasonably estimating the withdrawn parameters (see Table 2). Sensitivity analysis was then carried out on the entire parameter space over time. It is observed from the results that differences in stigma levels can substantially alter the overall prognosis of EVD in the population. In addition, we used time series plots to examine the effects of increasing or decreasing stigma on the number of infected and unsafely buried deceased individuals. The results show that increasing stigma leads to an increase in the number of infected stigmatised individuals. This is unlike the result obtained in^22^ where human behaviour led to a fall in EVD transmission rate. Instead of negative human behaviours like stigmatization which leads to an increase in disease transmission, the authors of^22^ considered behavior change that avoids contact with the virus and is motivated by disease incidence. Since stigmatized individuals refuse to seek hospital care, they are more likely to die from the disease and be buried unsafely. This increase is also observed in the time series plot results of $D_u$ for different values of stigmatisation rate. The model in^22^ did not look at burials separately. They assumed that all burials are safe since their focus was on positive human behavior, which is unlike the case of a typical outbreak: this may have a serious impact on their results. Dead bodies of Ebola deceased individuals are more infectious than the infected who are alive, hence an increase in the number of dead bodies with unsafe burials can be disastrous in the event of an outbreak. It follows that stigma is an important factor in the spread of EVD. We, therefore, recommend that EVD-control strategies should focus on the reduction of EVD-related stigma through a combination of targeted education about the disease, awareness campaigns and programs to re-integrate survivors into their communities. However these strategies are not sufficient, they may need to be supplemented by other control measures such as quarantine, increase in number of beds in Ebola treatment units, contact tracing, the use of protective equipment and vaccination to attain disease eradication.

The model proposed in this paper had one major limitation which when addressed, could create an opportunity to re-look at the model. The lack of sufficient data on the number of Ebola stigmatised infected individuals limited the fitting results and reduced the accuracy of the parameter estimation. This model can thus be improved by fitting it to data for stigmatised infected individuals to give a more accurate set of parameter values and simulation results. Also, the model does not necessarily give a complete picture of a typical Ebola outbreak as a typical Ebola outbreak consists of complex processes and occurrences that can hardly be incorporated into one mathematical model. For instance, While someone can be stigmatized if they are simply infected, incubating, and not yet subjectively or visibly ill, others in this stage may not. Our model only captures a scenario in which individuals who suspect that they have the virus by virtue of their exposure to it develop internal stigma which is usually seen in the way they hide even information about their contact with an infectious person. however, a typical outbreak is made of several other different scenarios. In a typical outbreak, a proportion of those who initially refuse to seek hospital care eventually change their minds and go to the hospital. We assumed that this number is negligible because the indigents of the North and south Kivu provinces of the DRC which are the case studies in this work are primitive and traditionally inclined people who so much hold onto their traditional beliefs and have very little trust in formal health care. This may have impacted our results in one way or the other. Also, most deceased bodies of individuals who die in the community are usually hiddenly buried by their relatives because of a belief that the spirit of the deceased person will later hunt them if not given a befitting burial. This scenario which is common in the North and south Kivu provinces of the DRC is the scenario that was captured in this model which is not in general so.

## Data Availability

The dataset analysed during the current study is available from the WHO website^42^.
